# Comparative Utility of Genetic Determinants of Drug Resistance and Phenotypic Drug Susceptibility Profiling in Predicting Clinical Outcomes in Patients With Multidrug-Resistant *Mycobacterium tuberculosis*

**DOI:** 10.3389/fpubh.2021.663974

**Published:** 2021-04-22

**Authors:** Yang Che, Tianchi Yang, Lv Lin, Yue Xiao, Feng Jiang, Yanfei Chen, Tong Chen, Jifang Zhou

**Affiliations:** ^1^Ningbo Municipal Center for Disease Control and Prevention, Institute of Tuberculosis Prevention and Control, Ningbo, China; ^2^School of International Pharmaceutical Business, China Pharmaceutical University, Nanjing, China

**Keywords:** genetic determinant, resistance, multidrug, mycobacterium tuberculosis, phenotypic drug susceptibility test

## Abstract

**Setting:** Programmatic management of drug-resistant tuberculosis in Ningbo, China.

**Objective:** To assess whether data-driven genetic determinants of drug resistance patterns could outperform phenotypic drug susceptibility testing in predicting clinical meaningful outcomes among patients with multidrug-resistant tuberculosis (MDR-TB).

**Design:** We conducted a prospective cohort study of 104 MDR-TB patients. All MDR-TB isolates underwent drug susceptibility testing and genotyping for mutations that could cause drug resistance. Study outcomes were time to sputum smear conversion and probability of treatment success, as well as time to culture conversion within 6 months. Data were analyzed using latent class analysis, Kaplan–Meier curves, and Cox regression models.

**Results:** We report that latent class analysis of data identified two latent classes that predicted sputum smear conversion with *P* = 0.001 and area under receiver-operating characteristic curve of 0.73. The predicted latent class memberships were associated with superior capability in predicting sputum culture conversion at 6 months and overall treatment success compared to phenotypic drug susceptibility profiling using boosted logistic regression models.

**Conclusion:** These results suggest that genetic determinants of drug resistance in combination with phenotypic drug-resistant tests could serve as useful biomarkers in predicting treatment prognosis in MDR-TB.

## Introduction

According to World Health Organization (WHO) report, there were approximately half a million new cases of rifampicin-resistant tuberculosis (TB) worldwide, of which 78% had multidrug-resistant TB in 2019. China is among the countries with the highest burden of multidrug-resistant tuberculosis (MDR-TB), with prevalence rate of MDR-TB at 5.7 and 25.6% among new and previously treated cases, respectively (1, 2).

Early detection of MDR-TB is essential to worldwide TB eradication efforts, as MDR-TB continues to be a great public health threat (3, 4). While drug susceptibility testing (DST) for culture-positive TB patients is recommended by the national guidelines on TB management, there is a growing evidence in the literature that phenotypic DST is not only time consuming but also oftentimes fails to detect mutations that confer poor clinical outcomes (5, 6). The effect of drug resistance on treatment outcomes has not been studied adequately. In addition, it is unclear if genotypic DST results could supplement first- and second-line phenotypic drug susceptibility profiling to better predict treatment outcomes among patients with MDR-TB.

We aimed to identify subgroups of MDR-TB patients who share similar patterns of genetic determinants of drug resistance using latent class analysis (LCA). We compared demographic and clinical characteristics associated with genotypic DST latent classification. Finally, we investigated the comparative utility of genetic mutation profile and the drug-resistant phenotype in predicting clinical important outcomes of MDR-TB.

## Materials and Methods

### Study Population

We conducted the present study in Ningbo, China, where the estimated incidence of TB was 43.77 per 100,000 residents in 2019. We conducted a prospective cohort study between 2015 and 2017 in Ningbo, China. All subjects were followed until cure, treatment completion, death, end-of-study period (31 December 2019), loss to follow-up, whichever came first. MDR-TB [defined as resistant to at least isoniazid (INH) and rifampin (RFP)] was identified by a laboratory at Ningbo Tuberculosis Control Institute using the conventional DST. Study participants signed an informed consent form and were enrolled. Their demographic characteristics and clinical information were obtained from patients' medical record at local TB dispensaries. Patients were included if they were diagnosed as MDR-TB and if they gave informed consent. Exclusion criteria included pregnancy, age below 18 years old at TB diagnosis, and serious liver or renal dysfunction.

This study was approved by the Ethics Committee of Ningbo Municipal Center for Disease Control and Prevention. Written informed consent was obtained from all participants. This study was conducted in accordance with the Declaration of Helsinki.

### Drug Susceptibility Testing and Strain Identification

The isolates were cultured on Lowenstein–Jensen (L–J) medium for 4–8 weeks, and the culture with growing colonies were further evaluated for drug susceptibility testing at regional reference laboratories. Testing for susceptibility to four first-line anti-TB drugs and seven second-line drugs was performed with the proportional method recommended by WHO (7). The concentrations of drugs in L–J medium were as follows: isoniazid (INH), 0.2 μg/ml; rifampicin (RFP), 40 μg/ml; ethambutol (EMB), 2 μg/ml; streptomycin (SM), 4 μg/ml; ofloxacin (OFX), 2 μg/ml; levofloxacin (LFX), 2 μg/ml; kanamycin (KM), 30 μg/ml; amikacin (AMK), 30 μg/ml; capromycin (CM), 40 μg/ml; protionamide (PTO), 40 μg/ml, and p-aminosalicylic acid (PAS), 1 μg/ml (7). The pyrazinamide (PZA) drug susceptibility testing was performed with a Bactec MGIT 960 system, and critical concentration was 100 μg/ml (8). Quality control was performed during DST using the H37RV reference strains. Members of the Beijing family of strains were identified by the RD105 multiplex PCR (9).

### DNA Extraction and Sequencing of Drug-Resistance-Related Genes

The crude DNA was extracted from freshly harvested bacteria as reported (10). The cultured bacteria that were extracted from the surface of L–J medium were suspended in 500 μl Tris-EDTA (TE) buffer and heated in a 95°C water bath for 30 min. The genomic DNA was used as template for amplification. Expected fragments were amplified. Each 30 μl PCR mixture contained 15 μl 2 × GoldStar MasterMix (CWBio, Beijing, China), 1 μl (each) of the forward and reverse primers (5 μM), 12 μl distilled H_2_O, and 1 μl of genomic DNA. The reaction conditions consisted of a denaturation step of 5 min at 95°C followed by 35 cycles of 30 s at 94°C, 30 s at 60°C, and 45 s at 72°C, with a final extension step of 5 min at 72°C. PCR products were carried out at Personalbio Company (Shanghai, China). Gene polymorphisms were aligned with pncA of reference strain H37RV (ATCC) using DNAstar MegAlign (version 7.1) software.

### Definitions of DR-TB Types and Outcomes

MDR-TB were defined as those resistant to both isoniazid and rifampicin. Pre-XDR TB was defined as MDR-TB additionally resistant to either quinolone family or second-line anti-TB injectable drugs, but not both. XDR-TB was defined as MDR-TB resistant to any member of the quinolone family and at least one of the remaining second-line anti-TB injectable drugs (11).

All patients were followed for the treatment outcomes. Sputum smear and culture were performed in accordance of the national TB guidelines. We assessed time to sputum conversion as time-to-event endpoint while time to culture conversion at month 6 after treatment initiation as binary outcomes. We further used standard WHO outcomes definitions for MDR tuberculosis: cure, treatment completion, treatment failure, death from any case, default, and transfer out (12, 13). We defined successful outcomes as cure or completion of treatment and poor outcomes as failure or death. Collectively, these were considered favorable outcomes, whereas unfavorable outcomes included default, transfer, or continuing treatment.

### Statistical Analysis

We performed latent class analysis models to estimate patterns of genotypic markers in the sample of MDR-TB isolates (14). Seven genetic determinants comprising *pnc*A, *rrs, rpsL, gyr*A, *gyr*B, *emb*B, and Beijing genotype were included as explanatory latent class indicators. Model fit indices such as Akaike Information Criterion (AIC) and Bayesian Information Criterion (BIC) were calculated to determine the best-fitting subclass structure, with smaller values indicating better fit (15). A series of models with increasing number of classes ranging from 2 to 7 were assessed to determine the optimal number of latent classes using model fit indices, interpretability, theoretical justification, and parsimony considerations. The final LCA model selection was based upon these measures varied within a plausible range and clinical judgment.

Descriptive and inferential statistics were used to compare baseline demographic and clinical characteristics across latent classes within MDR-TB cohort. Continuous variables were summarized as means and standard deviations. Categorical variables were summarized as counts and percentages. Statistical comparisons between groups were performed using Student's *t-*test for continuous variables and χ^2^ test for categorical variables.

Once the optimal number of latent classes was defined, we used univariate logistic regression models to estimate odds ratios (ORs) and 95% confidence intervals (CIs) for associations between latent class membership, baseline demographic, clinical characteristics, and DST-related covariates with binary outcomes. We used multivariable Cox regression models to estimate hazard ratios (HRs) and 95% CIs for associations between pretreatment characteristics and sputum smear conversion in time-to-event analysis.

We then assessed the performance of both genotypic latent class and phenotypic DST results as predictors in fully adjusted multivariable analyses on all three clinical outcomes. LogitBoost classification algorithm with built-in feature selection was applied on the analytical dataset using the popular open-source R package caret. In predictive model, the learning objective function was binary logistic. Overfitting was minimized by introducing log-likelihood loss function to reduce the sensitivity to noise and outliers. Its performance is further enhanced by performing classification via combining many weak classifiers into a more robust classifier (16). All classifiers were run on the dataset in a 10-repeated nested 5-fold cross-validation with hyperparameter tuning. Performance metrics [area under the curve (AUC) and accuracy] were computed.

All statistical tests were two-tailed, and a *p* < 0.05 was defined *a priori* as statistically significant. Analyses were conducted using open-source software R 4.0.2 including the poLCA and caret packages (https://CRAN.R-project.org).

## Results

### Demographic and Clinical Characteristics of Study Participants

**Figure 1:**
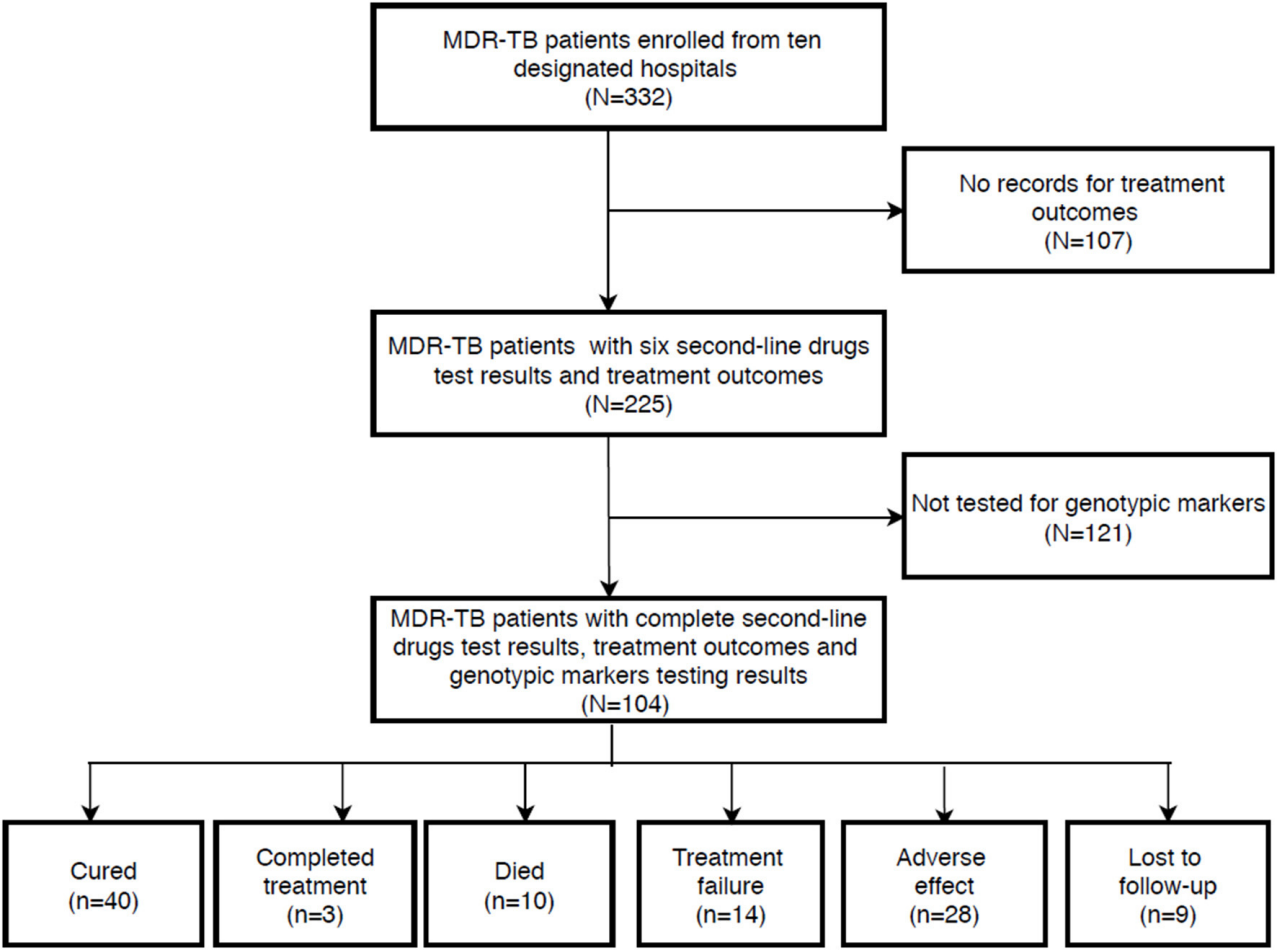
Schematic presentation of patient flow diagram.

During the study period, a total of 332 patients were diagnosed with MDR-TB, of which 225 consecutive MDR-TB patients with full medical and microbiological information were assessed for study eligibility. After excluding patients who failed to meet the selection criteria, 104 MDR-TB patients with complete genotypic markers profiles were included in the final analysis (Figure 1).

The baseline characteristics for the eligible patients with MDR-TB according to genetic determinants of drug resistance patterns are presented in Table 1. The majority of patients were male (71.2%), permanent resident (54.8%), and have a previous history of tuberculosis treatment (61.5%). Cavity disease was present in 60 patients (57.7%). With regard to the treatment history among MDR-TB cases, the percentage of retreated MDR-TB patients in the class 2 group was significantly higher than in the class 1 group (*P* < 0.05). Additionally, we found that no statistically significant difference between the class 1 and class 2 group in age, gender, permanent resident, and cavity. Model fit statistics are shown in Supplementary Table 1.

**Table 1:** Baseline characteristics for the eligible patients with MDR-TB according to genetic determinants of drug resistance patterns.

**Patient characteristic**	**Overall of isolates**		**No. (%) of isolates class 1**		**No. (%) of isolates class 2**		***P-*value**
	***N***	**%**	***N***	**%**	***N***	**%**	
	***N =* 104**		***N =* 47**		***N =* 57**		
Male sex	74	71.2%	35	74.5%	39	68.4%	0.50
Female sex	30	28.8%	12	25.5%	18	31.6%	
**Age at TB diagnosis**							
(Median, IQR)	44	33–59	43	33–59	45	33–59	0.66
Permanent resident	57	54.8%	27	57.4%	30	52.6%	0.62
Treatment history							
New case	40	38.5%	10	21.3%	30	52.6%	<0.01
Re-treated	64	61.5%	37	78.7%	27	47.4%	
Cavity	60	57.7%	28	59.6%	32	56.1%	0.72
***M. tuberculosis*** **type**							
*Beijing family*	83	79.8%	37	78.7%	46	80.7%	
Non-*Beijing* family	21	20.2%	10	21.3%	11	19.3%	0.80
PncA gene mutation	53	51.0%	45	95.7%	8	14.0%	0.00
rrs gene mutation	8	7.7%	5	10.6%	3	5.3%	0.31
rpsL gene mutation	52	50.0%	29	61.7%	23	40.4%	0.03
gyrA gene mutation	35	33.7%	30	63.8%	5	8.8%	<0.01
gyrB gene mutation	3	2.9%	2	4.3%	1	1.8%	0.87
embB gene mutation	59	56.7%	41	87.2%	18	31.6%	<0.01
MDR	104	100.0%	47	100.0%	57	100.0%	
Pre-XDR	31	29.8%	26	55.3%	5	8.8%	<0.01
XDR	6	5.8%	4	8.5%	2	3.5%	0.51
**Resistance to**							
SM	61	58.7%	32	68.1%	29	50.9%	0.08
EMB	49	47.1%	27	57.4%	22	38.6%	0.06
OFX	31	29.8%	27	57.4%	4	7.0%	<0.01
LFX	31	29.8%	26	55.3%	5	8.8%	<0.01
KM	8	7.7%	5	10.6%	3	5.3%	0.51
AMK	7	6.7%	4	8.5%	3	5.3%	0.79
CM	3	2.9%	0	0.0%	3	5.3%	0.31
PTO	5	4.8%	3	6.4%	2	3.5%	0.83
PAS	20	19.2%	8	17.0%	12	21.1%	0.60
**Drug resistance pattern**							
SM, INH, RFP, and EMB	32	30.8%	21	44.7%	11	19.3%	0.01
INH and RFP	26	25.0%	9	19.1%	17	29.8%	0.21
INH, RFP, and SM	29	27.9%	11	23.4%	18	31.6%	0.36
INH, RFP, and EMB	17	16.3%	6	12.8%	11	19.3%	0.37
**Treatment outcomes**							
Sputum negative at month 2	76	73.1%	33	70.2%	43	75.4%	0.55
Sputum negative at month 6	76	73.1%	31	66.0%	45	78.9%	0.14
Culture negative at month 6	76	73.1%	31	66.0%	45	78.9%	0.14
Sputum converted to negative	82	78.8%	34	72.3%	48	84.2%	0.14
Cured	40	38.5%	16	34.0%	24	42.1%	0.40
Treatment completed	3	2.9%	1	2.1%	2	3.5%	1.00
Treatment success	43	41.3%	17	36.2%	26	45.6%	0.33
Death	10	9.6%	5	10.6%	5	8.8%	0.75
Treatment interrupted due to ADR	42	40.4%	19	40.4%	23	40.4%	0.99
Lost to follow-up	9	8.7%	6	12.8%	3	5.3%	0.18

*IQR, inter quantile range; INH, isoniazid; RFP, rifampicin; SM, streptomycin; EMB, ethambutol; OFX, ofloxacin; LFX, levofloxacin; KM, kanamycin; AMK, amikacin; CM, capreomycin; PTO, protionamide; PAS, paza-aminosalioylate; MDR, multi drug resistance; Pre-XDR, pre-extensively extensive drug resistance; XDR, extensive drug resistance*.

### Characteristics of Genotypic Markers

Totally, 53 out of 104 (51.0%) MDR-TB isolates observed a mutation located in the *pnc*A gene, including 48 (90.6%, 48/53) of single nucleotide substitutions and 5 (9.4%, 5/53) of frame-shift mutation. As shown in Supplementary Table 2, we found a great mutant diversity in *pnc*A gene (17–19). The gene mutation percentage of *rrs, rpsL, gyrA, gyrB*, and *embB* in MDR-TB isolates were (7.7%, 8/104), (50.0%, 52/104), (33.7%, 35/104), (2.9%, 3/104), and (56.7%, 59/104). We found the gene mutation percentage of *pnc*A, *rps*L, *gyr*A, and *emb*B in class 1 group was significantly higher than in class 2 group (P < 0.05) (Supplementary Figures 1, 2).

### Drug Susceptibility Profiles

We analyzed the resistance phenotypic of anti-TB drugs between class 1 and class 2 groups. We found that the resistance of ofloxacin (57.4 vs. 7.0%, *P* < 0.001), levofloxacin (55.3 vs. 8.8%, *P* < 0.001), and pre-XDR (55.3 vs. 8.8%, *P* < 0.001) were more frequently detected among class 1 groups compared with class 2 groups. Additionally, we also found that the drug resistance pattern (INH + RFP + SM + EMB) has significant difference between class 1 and class 2.

### Treatment Outcomes

Successful treatment outcome occurred in 43 (41.3%) of the 104 patients. Forty-two (40.4%) treatment interrupted due to ADR, 10 (9.6%) died, and 9 (8.7%) lost to follow-up. It showed that no statistically significant difference between the class 1 and class 2 group in treatment outcome. Among the sputums of 104 patients, 76 (73.1%) were negative at 2 months, 76 (73.1%) negative at 6 months, and 82 (78.8%) converted to negative. There was no significant difference between class 1 and class 2 in sputum conversion at 2 or 6 months and no difference in culture conversion at 6 months after TB diagnosis.

In this study, MDR-TB treatment was administered daily, in accordance with WHO guidelines. The recommended treatment course comprises a 6-month intensive phase and 18-month continuation phase using the regimen: 6ZKmLfxPtoPAS (Z = PZA; Km = Kanamycin, Lfx = levofloxacin; Pto = ethionamide; PAS = para-aminosalicylic acid).

### Factors Related With Sputum Conversion, Treatment Success, and 6-Month Sputum Culture Conversion

**Figure 2:**
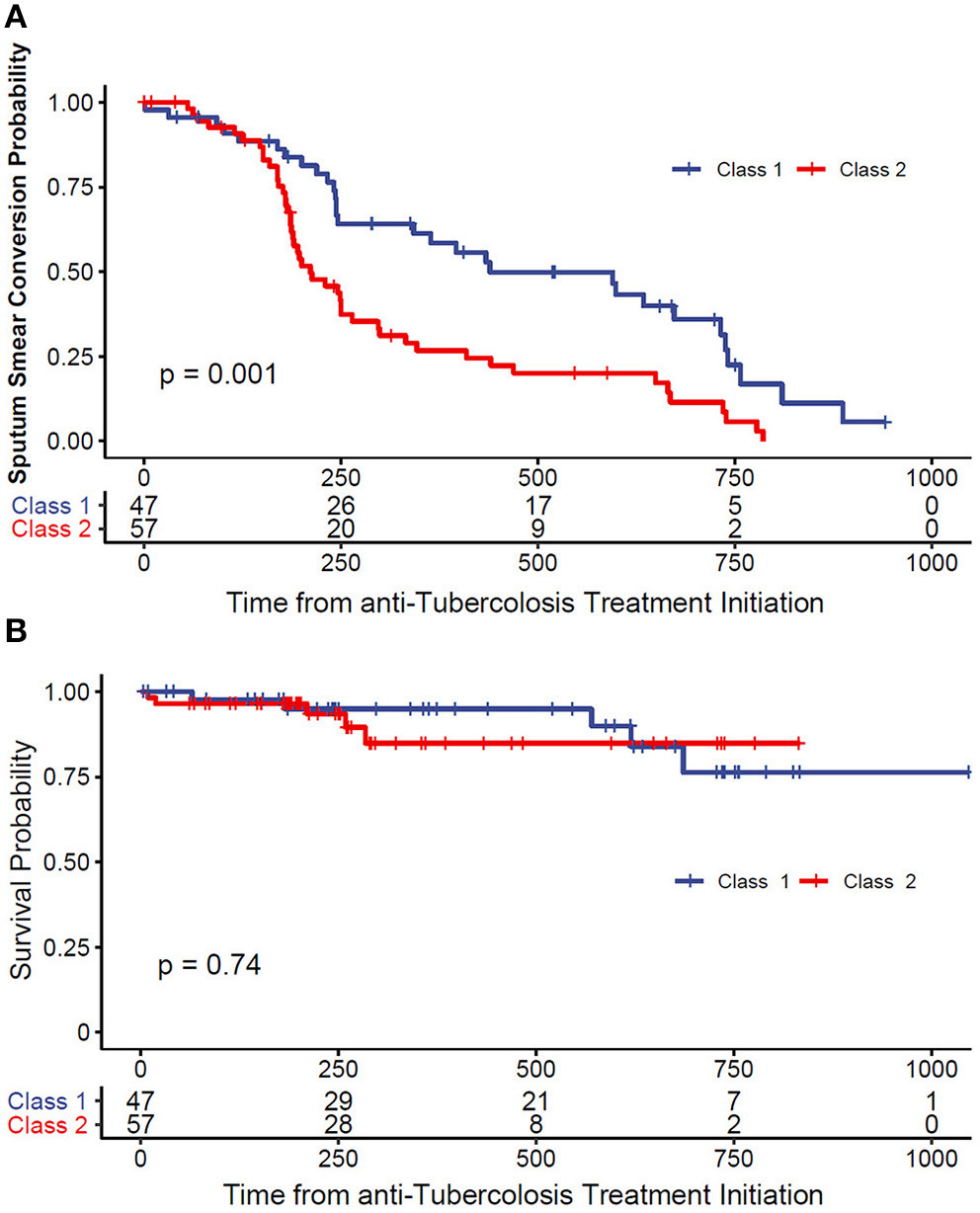
**(A)** Kaplan–Meier survival curve of time to smear conversion. **(B)** Kaplan–Meier survival curve of all-cause mortality.

As showed in Table 2, in univariate analysis, the latent class membership was associated with overall sputum smear conversion (HR = 2.18; 95% CI, 1.36–3.50). While our results failed to confirm statistically significant association between overall treatment success, in relation to LCA membership (OR = 1.48; 95% CI, 0.67–3.26), we failed to demonstrate statistically significant association between LCA membership and culture conversion by the end of 6 months from TB diagnosis (OR = 2.18; 95% CI, 0.87–5.47, *p* = 0.10). We also found that *pnc*A, *rpsL*, and *gyr*A mutations were associated with poor clinical outcomes. As shown in Figure 2, the time-to-event analyses evaluated the association between LCA membership and meaningful clinical outcomes.

**Table 2:** Univariate association between molecular/phenotypic drug susceptibility tests findings and clinical outcomes.

	**Sputum smear conversion (HR, 95%CI)**	***P***	**Treatment success (OR, 95%CI)**	***P***	**Culture conversion at month 6 (OR, 95%CI)**	***P***
Latent class membership 2 vs. 1	2.18 (1.36–3.50)	<0.01	1.48 (0.67–3.26)	0.33	2.18 (0.87–5.47)	0.10
*pncA* mutation	0.55 (0.35–0.87)	0.01	0.74 (0.34–1.61)	0.45	0.51 (0.20–1.29)	0.15
*rrs* mutation	0.55 (0.20–1.51)	0.25	1.46 (0.35–6.20)	0.61	0.29 (0.07–1.27)	0.10
*rpsL* mutation	0.55 (0.34–0.86)	0.01	0.57 (0.26–1.26)	0.17	0.48 (0.19–1.22)	0.12
*gyrA* mutation	0.52 (0.32–0.86)	0.01	1.31 (0.58–2.98)	0.52	0.69 (0.27–1.76)	0.44
*gyrB* mutation	1.44 (0.35–5.96)	0.61	0.000 (0.000)	1.00	0.65 (0.06–7.47)	0.73
*embB* mutation	0.60 (0.38–0.95)	0.03	0.94 (0.43–2.06)	0.87	0.82 (0.33–2.07)	0.68
Beijing genotype	1.14 (0.63–2.09)	0.66	0.93 (0.35–2.44)	0.88	1.11 (0.36–3.46)	0.86
Phenotypic DST tests SM	1.64 (1.04–2.59)	0.03	0.70 (0.32–1.54)	0.37	0.48 (0.18–1.28)	0.14
EMB	1.30 (0.82–2.05)	0.26	0.70 (0.32–1.53)	0.37	0.64 (0.26–1.58)	0.33
KM	1.16 (0.42–3.20)	0.77	0.45 (0.09–2.33)	0.34	0.29 (0.07–1.27)	0.10
CM	0.28 (0.08–0.92)	0.04	1.07 (0.23–5.04)	0.93	0.41 (0.09–1.96)	0.26
OFX	1.34 (0.81–2.24)	0.26	1.25 (0.54–2.92)	0.61	0.87 (0.33–2.30)	0.77
LFX	1.40 (0.84–2.31)	0.20	1.04 (0.44–2.43)	0.94	0.87 (0.33–2.30)	0.77
MFX	1.29 (0.62–2.72)	0.50	0.94 (0.25–3.56)	0.93	1.35 (0.27–6.84)	0.72
PTO	1.16 (0.36–3.69)	0.80	0.94 (0.15–5.90)	0.95	0.99 (0.10–9.93)	0.99
PAS	1.23 (0.71–2.15)	0.46	1.99 (0.74–5.32)	0.17	0.98 (0.32–3.05)	0.98

*HR, hazard ratio; CI, confidence interval; OR, odds ratio; DST, drug susceptibility test; SM, streptomycin; EMB, ethambutol; KM, kanamycin; CM, capreomycin; OFX, ofloxacin; LFX, levofloxacin; MFX, moxifloxacin; PTO, protionamide; PAS, paza-aminosalioylate*.

### Association Between Latent Class Membership and Outcomes

After adjusting for potential confounding factors based on multivariable regression models, we found that latent class memberships were positively associated with sputum smear conversion (AUC-ROC = 0.73), compared to the model using phenotypic DST findings (AUC-ROC = 0.69). On the other hand, the LCA membership performed better than the phenotypic DST in predicting overall treatment success (AUC-ROC 0.63 vs. 0.57) (Supplementary Figure 3).

## Discussion

We performed a latent class analyses in predicting clinical meaningful outcomes among patients with MDR-TB. We developed and evaluated the association between latent class membership and treatment outcomes at 2 and 6 months following systemic TB treatment. We found that the latent class membership based on genetic features outperformed traditional DST approach in predicting sputum conversion events.

To our knowledge, we were among the few investigators using LCA in evaluating prognosis of MDR-TB patients in combination with parallel culture-based DST findings, using over 2 years follow-up data in the majority of patients. After adjusting for potential confounding factors, we found that the LCA-derived membership status was statistically significantly associated with overall sputum conversion events. However, due to limited sample size, our analysis was underpowered to conclusively demonstrate association between LCA membership and overall treatment success at the standard alpha level of 0.05.

Our results are in agreement with those from preexisting reports. We found that ~17% of patients with MDR-TB had discrepancies between molecular and phenotypic DST tests for susceptibility to PZA treatment. A recent study in China demonstrated the usefulness of *pncA* gene mutation in predicting clinical outcomes, when combined with clinical information such as treatment regimen and age (20). Other multinational cohort studies suggested that *gyrA* mutations were associated with increased mortality risks among patients with MDR/XDR-TB (21, 22). However, these studies were limited in restricting molecular tests findings to single gene mutations and failed to evaluate predictive models using all available genetic test results in a comprehensive manner. Using our data-driven approach, we were capable of combining important clinical characteristics extracted from TB registries and medical charts to build user-friendly clinical decision support tools.

Our study has a number of important policy and clinical implications worth mentioning. Prognosis prediction is challenging in MDR-TB management, and DST evaluation remains problematic due to long turnaround time. While confirmatory drug resistance profiling based on genetic tests is time saving and less ambiguous, the multiple combinations of molecular mutation status prohibits more straightforward evaluation of DST findings.

Despite the fact that our LCA model has been carefully designed, we acknowledge that our results might be limited, as the interpretation of these findings depends on a number of assumptions. One of the strength of our study was that we used treatment outcomes to validate the results of both phenotypic and genotypic DST to directly compare the predictive values of the two approaches. Since poor prognosis is associated with other risk factors such as HIV infection, malnutrition, medication adherence, and other comorbidities not routinely measured in clinical practice, this explains the imperfect correlation between LCA membership and *in vitro* susceptibility tests. Here, our data-driven LCA models achieved more accurate prognosis prediction in overall sputum conversion, highlighting the clinical utility of this method.

Future study should aim to further improve the performance of molecular testing by incorporating mode detailed specific mutations of genes that are confirmed to associate with poor outcomes. We also hypothesize that inclusion of more host-related socioeconomic risk factors for poor clinical outcomes could improve the accuracy of prognostic prediction among MDR-TB patients.

## Data Availability Statement

The raw data supporting the conclusions of this article will be made available by the authors, without undue reservation.

## Ethics Statement

The studies involving human participants were reviewed and approved by Ethics Committee of Ningbo Municipal Center for Disease Control and Prevention. The patients/participants provided their written informed consent to participate in this study.

## Author Contributions

YC conceived the study and was involved in the design, analysis, report writing, and drafting the manuscript. TY, LL, YX, FJ, YChe, and TC were involved in the conception, design, and supervised the work. JZ was involved in manuscript review. All authors contributed to and approved the final draft.

## Conflict of Interest

The authors declare that the research was conducted in the absence of any commercial or financial relationships that could be construed as a potential conflict of interest.
