# Supplementary material for: Comparative Utility of Genetic Determinants of Drug Resistance and Phenotypic Drug Susceptibility Profiling in Predicting Clinical Outcomes in Patients With Multidrug-Resistant Mycobacterium tuberculosis
Source: Front Public Health. 2021 Apr 22;9:663974. doi: 10.3389/fpubh.2021.663974 (PMC8100237; doi:10.3389/fpubh.2021.663974)
Supplement: Supplementary file 2 [file Data_Sheet_1.docx]

Supplementary Material

**Supplemental Table 1**. **Model fit statistics**

| **Number of classes** | **AIC** | **BIC** | **Likelihood ratio/deviance statistic** | **Chi-square goodness of fit** | **Selected** |
| --- | --- | --- | --- | --- | --- |
| 2 | 743.873 | 783.539 | 67.6729 | 75.1534 | √ |
| 3 | 738.331 | 799.152 | 46.131 | 92.5894 |  |
| 4 | 746.129 | 828.106 | 37.9293 | 36.1715 |  |
| 5 | 746.388 | 849.519 | 22.1876 | 18.1899 |  |
| 6 | 758.152 | 882.438 | 17.9517 | 14.4075 |  |
| 7 | 772.301 | 917.743 | 16.1011 | 13.9335 |  |

Abbreviations: AIC, Akaike information criterion; BIC, Bayesian information criteria

**Supplemental Table 2** **Mutations of *PncA* gene among MDR-TB isolates**

| **Nucleotide position** | **Codon Change** | **A.A change** | **Mutation Type** | **TBDReaMDB** | **GMTV Database** | **No. of isolates** |
| --- | --- | --- | --- | --- | --- | --- |
| T14>G | 5. ATC/AGC | ILE5SER | Non-synonymous | Unreported | Unreported | 2 |
| T17>C | 6. ATC/ACC | ILE6THR | Non-synonymous | Unreported | Reported | 2 |
| G19>T | 7.GTC/TTX | VAL7PHE | Non-synonymous | Reported | Reported | 1 |
| A23>G | 8. GAC/GGC | ASP8GLY | Non-synonymous | Reported | Reported | 1 |
| A29>C | 10. CAG/CCG | GLN10PRO | Non-synonymous | Reported | Reported | 2 |
| A35>C | 12. GAC/GCC | ASP12ALA | Non-synonymous | Reported | Reported | 1 |
| T40>G | 14. TGC/GGC | CYS14GLY | Non-synonymous | Unreported | Unreported | 1 |
| T56>G | 19.CTG/CGG | LEU19ARG | Non-synonymous | Reported | Unreported | 1 |
| G71>A | 24. GGC/GAC | GLY24ASP | Non-synonymous | Unreported | Reported | 1 |
| T100>G | 34. TAC/GAC | TYR34/ASP | Non-synonymous | Unreported | Unreported | 1 |
| A139>G | 47. ACC/GCC | THR47ALA | Non-synonymous | Reported | Reported | 1 |
| A142>G | 48. AAG/GAC | LYS48GLU | Non-synonymous | Reported | Reported | 1 |
| C151>T | 51. CAC/TAC | HIS51TYR | Non-synonymous | Reported | Reported | 2 |
| A170>C | 57. CAC/CCC | HIS57PRO | Non-synonymous | Reported | Unreported | 1 |
| C184>A | 62. CCG/ACG | PRO62THR | Non-synonymous | reported | Unreported | 1 |
| C206>G | 69. CCA/CGA | PRO69ARG | Non-synonymous | Unreported | Unreported | 1 |
| A226>C | 76. ACT/CCT | THR76PRO | Non-synonymous | Reported | Reported | 1 |
| A287>C | 96. AAG/ACG | LYS96/THR | Non-synonymous | Reported | Reported | 1 |
| 294-95 | Deletion T | FRAMSHIPT | FRAMSHIPT | Unreported | Unreported | 1 |
| C312>A | 104. AGC/AGA | SER104ARG | Non-synonymous | Reported | Reported | 1 |
| G314>A | 105. GGC/GAC | GLY105ASP | Non-synonymous | Reported | Reported | 1 |
| A329>G | 110. GAC/GGC | ASP110GLY | Non-synonymous | reported | Unreported | 1 |
| A345>C | 115. CCA/CCC | PRO115PRO | Synonymous | Unreported | Unreported | 1 |
| T347>G | 116. CTG/CGG | LEU116ARG | Non-synonymous | Reported | Reported | 1 |
| C372>A | 124. GGC/GGA | GLY124GLY | Synonymous | Unreported | Unreported | 1 |
| T374>G | 125. GTC/GGC | VAL125GLY | Non-synonymous | Reported | Reported | 1 |
| 392-93 | Insertion GG | FRAMSHIPT | FRAMSHIPT | Unreported | Unreported | 3 |
| G394>T | 132. GGT/TGT | GLY132CYS | Non-synonymous | reported | Unreported | 1 |
| C401>T | 134. GCC/GTC | ALA134VAL | Non-synonymous | Reported | Reported | 1 |
| A403>C | 135. ACC/CCC | THR135PRO | Non-synonymous | Reported | Reported | 1 |
| A407>C | 136. GAT/GCT | ASP136ALA | Non-synonymous | Unreported | Reported | 2 |
| A410>C | 137. CAT/CCT | HIS137PRO | Non-synonymous | Unreported | Unreported | 3 |
| G415>C | 139. GTG/CTG | VAL139LEU | Non-synonymous | reported | Unreported | 1 |
| T416>G | 139. GTG/GGG | VAL139ALA | Non-synonymous | Reported | Reported | 2 |
| A424>G | 142. ACG/GCG | THR142ALA | Non-synonymous | reported | Unreported | 3 |
| T464>G | 155. GTG/GGG | VAL155GLY | Non-synonymous | Reported | Reported | 1 |
| T470>G | 157. GTG/GGG | VAL157GLY | Non-synonymous | Unreported | Unreported | 1 |
| A478>C | 160. ACA/CCA | THR160PRO | Non-synonymous | Unreported | Reported | 2 |
| 497-98 | Insertion G | FRAMSHIPT | FRAMSHIPT | Unreported | Unreported | 1 |
| G538>T | 180. GTC/TTC | VAL180PHE | Non-synonymous | Reported | Unreported | 1 |

Abbreviations: MDR, multi drug resistance; TBDReaMDB, Tuberculosis Drug Resistance Mutation Database; GMTV, Genome-wide *Mycobacterium tuberculosis* variation

**Supplemental Figure 1: Distribution of genetic mutations by latent classes**

**Supplemental Figure 2: Probability of latent classes clustering**

**Supplemental Figure 3A** ROC curve analysis of models in predicting overall treatment success

**Figure 3 B** ROC curve analysis of models in predicting for overall sputum smear conversion
